# Acute Rho-kinase inhibition improves coronary dysfunction *in vivo*, in the early diabetic microcirculation

**DOI:** 10.1186/1475-2840-12-111

**Published:** 2013-08-01

**Authors:** James T Pearson, Mathew J Jenkins, Amanda J Edgley, Takashi Sonobe, Mandar Joshi, Mark T Waddingham, Yutaka Fujii, Daryl O Schwenke, Hirotsugu Tsuchimochi, Misa Yoshimoto, Keiji Umetani, Darren J Kelly, Mikiyasu Shirai

**Affiliations:** 1 Department of Physiology, Monash University, Melbourne, Australia; 2 Monash Biomedical Imaging Facility, Melbourne, Australia; 3 Australian Synchrotron, Melbourne, Australia; 4 Department of Medicine, St Vincent’s Hospital, University of Melbourne, Melbourne, Australia; 5 National Cerebral and Cardiovascular Center Research Institute, Suita, Japan; 6 The Ritchie Centre, Monash Institute of Medical Research, Melbourne, Australia; 7 Department of Physiology, Otago University, Dunedin, New Zealand; 8 Japan Synchrotron Radiation Research Institute, Harima, Japan

## Abstract

**Objectives:**

Activation of RhoA/Rho-kinase (ROCK) is increasingly implicated in acute vasospasm and chronic vasoconstriction in major organ systems. Therefore we aimed to ascertain whether an increase in ROCK activity plays a role in the deterioration of coronary vascular function in early stage diabetes.

**Methods:**

Synchrotron radiation microangiography was used to determine in vivo coronary responses in diabetic (3 weeks post streptozotocin 65 mg/kg ip) and vehicle treated male Sprague–Dawley rats (n = 8 and 6). Changes in vessel number and calibre during vasodilator stimulation before and after blockade of nitric oxide synthase and cyclooxygenase were compared between rats. Acute responses to ROCK inhibitor, fasudil (10 mg/kg iv) was evaluated. Further, perivascular and myocardial fibrosis, arterial intimal thickening were assessed by histology, and capillary density, nitrotyrosine and ROCK1/2 expressions were evaluated by immunohistochemical staining.

**Results:**

Diabetic rats had significantly elevated plasma glucose (*P* < 0.001 vs control), but did not differ in fibrotic scores, media to lumen ratio, capillary density or baseline visible vessel number or calibre. Responses to acetylcholine and sodium nitroprusside stimulation were similar between groups. However, in comparison to control rats the diabetic rats showed more segmental constrictions during blockade, which were not completely alleviated by acetylcholine, but were alleviated by fasudil. Further, second order vessel branches in diabetic rats were significantly more dilated relative to baseline (37% vs 12% increase, *P* < 0.05) after fasudil treatment compared to control rats, while visible vessel number increased in both groups. ROCK2 expression was borderline greater in diabetic rat hearts (*P* < 0.053).

**Conclusions:**

We found that ahead of the reported decline in coronary endothelial vasodilator function in diabetic rats there was moderate elevation in ROCK expression, more widespread segmental constriction when nitric oxide and prostacyclin production were inhibited and notably, increased calibre in second and third order small arteries-arterioles following ROCK inhibition. Based on nitrotyrosine staining oxidative stress was not significantly elevated in early diabetic rats. We conclude that tonic ROCK mediated vasoconstriction contributes to coronary vasomotor tone in early diabetes.

## Introduction

Diabetes is associated with coronary microvascular dysfunction due to an inability of the endothelium to maintain vasodilatory tone both at rest [1] and during stress [2,3]. This previous work has primarily focused on later time-points of diabetes, where microvascular damage is already well developed and thus difficult to reverse. As diabetes progresses these individuals are subjected to a vastly increased risk of ischaemic heart disease, acute myocardial infarction, and stroke [4,5]. Utilising synchrotron radiation (SR) microangiography, we have now demonstrated that even in the early stages of diabetes in the coronary circulation focal and segmental constrictions occur when prostacyclin and nitric oxide contribution is prevented, although globally basal endothelium-dependent vasodilation is maintained [6]. This impairment in vasodilatory capacity may contribute to the increased vulnerability to ischemia and myocardial infarction observed in advanced diabetes. Thus, it is of paramount importance that the mechanisms underlying this localised dysfunction are further elucidated, at a time point where potential intervention remains possible.

One pathway that is increasingly recognised to be involved in the pathogenesis of cardiovascular disease is the RhoA/ROCK pathway, which has been implicated in the progression of conditions including hypertension [7], stroke [8], coronary vasospasm and angina [9,10], ischemia-reperfusion injury and heart failure [11,12]. RhoA is a small plasma membrane bound guanosine-5'-triphosphate-binding protein, which when stimulated, activates ROCK [12]. It is thought that upregulation of RhoA/ROCK in the diabetic vasculature [13] causes subsequent phosphorylation of downstream signalling targets including myosin light chain phosphatase, and increased constriction of vascular smooth muscle [14-16]. Thus there may be a role for ROCK in the early diabetic coronary dysfunction we have previously described [6].

Acute treatment with ROCK inhibitors has shown promising results, with reduction in ischaemic damage [15,17-19], improvement in cerebral vasodilation in type II diabetic mice [20] and decreased vascular resistance and increased peripheral blood flow in patients with heart failure [11]. Notably, ROCK inhibitors may also have beneficial outcomes in preventing the development of coronary dysfunction, most likely by promoting or maintaining increased expression and activity of vasoprotective endothelial nitric oxide synthase (NOS) [14,21]. This study therefore aimed to ascertain whether ROCK plays a role in the deterioration of coronary vascular function in early stage diabetes.

## Methods

### Animals and experiments at the synchrotron

Experiments were conducted at SPring-8, Japan Synchrotron Radiation Research Institute, Hyogo, Japan with approval from the Animal Experiment Review Committee in accordance with the guidelines of the Physiological Society of Japan. Male Sprague Dawley rats (Japan SLC, Kyoto, Japan, 7 wks old) received either a vehicle injection of sodium citrate (0.1 M, pH 4) (control) or streptozotocin (STZ; 65 mg/kg i.p) to induce type I diabetes. All rats were on a 12 hr light/dark cycle at 18-25°C and were provided with food and water *ad libitum*. Three weeks after vehicle or STZ injection all rats underwent terminal angiography experiments. Fasted conscious blood glucose was measured via the tail vein two days prior to imaging to minimise stress.

### Experimental preparation

Under sodium pentobarbital anaesthesia (50 mg/kg i.p.), rats were intubated, artificially ventilated (Shinano, Tokyo, Japan; 40% oxygen) and the right carotid artery cannulated with a radiopaque 20-gauge BD Angiocath catheter (Becton Dickinson, Inc., Sandy, Utah, USA), placing the tip at the entrance of the aortic valve. Body temperature was maintained at 37°C, using a rectal thermistor coupled with a thermostatically controlled heating pad. Anaesthesia level was maintained via additional intraperitoneal boluses of pentobarbital (25 mg/kg/h). Sodium lactate (Sigma-Aldrich Japan K.K., Tokyo, Japan) was administered intravenously via the right jugular vein to maintain body fluids (3.0 ml/hr). A 0.3 ml sample of venous blood was collected in EDTA coated tubes, centrifuged at 4°C for 15 min at 5000 rpm and plasma removed for storage at −20°C until subsequent triglyceride concentration determination (SRL Inc., Tachigawa, Tokyo, Japan). A catheter filled with heparinised saline (12 units/ml), was inserted into the right femoral artery to record arterial pressure via a pressure transducer (MLT0699, AD Instruments, NSW, Australia) using CHART software (v5.4.1, AD Instruments, NSW, Australia) to determine mean arterial pressure (MAP) and heart rate (HR), simultaneous with recordings of the camera trigger over the cardiac cycle.

### Angiography protocol

Each rat was then placed in line with the horizontal X-ray beam and SATICON detector system (Hitachi Denshi Techno-system, Ltd., Tokyo, Japan and Hamamatsu Photonics, Shizuoka, Japan), as described previously [6]. Pancuronium bromide (Mioblock; 2 mg/kg, Sankyo, Tokyo, Japan,) was administered for neuromuscular blockade to prevent spontaneous breathing when artificial ventilation was briefly stopped during imaging. Iodinated contrast medium (Iomeron 350; Bracco-Eisai Co. Ltd, Tokyo, Japan) was injected intrarterially as a bolus (0.3 – 0.5 ml at 0.4 ml/s) into the aorta with a clinical autoinjector (Nemoto Kyorindo, Tokyo, Japan) at the start of image recording scans. At least 10 minutes was allowed for renal clearance of contrast between imaging scans. During each cine-scan, monochromatic X-rays at 33.2 keV (energy bandwidth 20–30 eV) and a flux ~10^10^ photons/mm^2^/s, passed through the rats chest and were recorded on the SATICON detector at 30 frames/s at 10-bit resolution for ~3 s intervals. For each cine-scan, 100 frames were recorded with a short shutter open time of 1.5-2.0 ms/frame and a 9.5 μm equivalent pixel size for the 9.5 × 9.5 mm input field with images stored in 1024 × 1024 pixel format.

### Experimental protocol

Endothelium-dependent and –independent vasodilatory responses were recorded in control (n = 6) and diabetic (n = 8) animals. Angiogram series were recorded at the end of 5 minute infusions of vehicle (sodium lactate 3.0 ml/hr), ACh (3.0 μg/kg/min), sodium nitroprusside (SNP 3.0 μg/kg/min), during vehicle infusion 30 minutes after combined inhibition of nitric oxide and prostacyclin production with Nω-nitro-l-arginine methyl ester (L-NAME, 10 mg/kg iv. bolus) and sodium meclofenamate (2 mg/kg iv. bolus) respectively. For simplicity, combined blockade refers to L-NAME + meclofenamate treatment together. Endothelium-dependent vasodilation was then assessed during combined blockade with a repeat infusion of ACh (3.0 μg/kg/min). A final image series was recorded 10 minutes after administration of fasudil hydrochloride (10 mg/kg iv. ROCK inhibitor, HA1077, Tocris) [22]. Hence, all rats were imaged during 6 consecutive treatment periods in total.

### Tissue collection histology and immunohistochemistry

Hearts were fixed in 10% neutral buffered formalin and stored in 70% ethanol. All histological and immunohistochemical sections were imaged using the Aperio ScanScope XT Slide Scanner (Aperio Technologies, Inc., CA, USA) system. The proportional area of the stained protein was automatically quantified using the Positive Pixel Count v9 algorithm on Aperio Imagescope (v11.0.2.725, Aperio Technologies). Non-round vessels, resulting from oblique transection or branching, were excluded from quantification of fibrosis and media-to-lumen ratio.

In 4 μm thick sections of LV the vessel media-to-lumen ratio (the area of the vessel media wall divided by the area of the total blood vessel lumen) was calculated [21]. Myocardial interstitial and perivascular fibrosis was assessed using picrosirius red stained LV sections [6]. Perivascular fibrosis was evaluated around coronary arterioles, as the ratio of the area of fibrosis immediately surrounding the intramyocardial blood vessel walls to the total area of the vessel [21].

Capillary density in the myocardium was detected as the proportion of positively stained endothelial cells with murine-specific endothelial cell marker isolectin B4 (1:50, Vector Laboratories, Inc, Burlingame, CA, USA) [23]. Briefly, after dewaxing and heat-mediated antigen retrieval, nonspecific protein binding was blocked with 20% normal goat serum (Dako, Golstrup, Denmark). Sections were then incubated with biotinylated isolectin B4 at 4°C over-night, followed by avidin-biotin horseradish peroxidase (Vector Laboratories, Inc, Burlingame, CA, USA) and diaminobenzidine (Vector Laboratories, Inc, Burlingame, CA, USA), as described previously [23].

ROCK1/2 and nitrotyrosine staining was performed after subjecting sections to heat-mediated antigen retrieval, followed by incubation with 3% H_2_O_2_ for 15 min at room temperature and washing three times with PBS (pH 7.4) for 5 min each. Nonspecific protein binding was blocked with 20% normal goat serum (Dako, Golstrup, Denmark) for 30 min. The sections were then incubated with the primary antibody overnight at 4°C (ROCK1 1:200 dilution, and ROCK2 1:250 dilution, Abcam, Cambridge, USA; anti-nitrotyrosine 1:400, Millipore 6–284). Following this, sections were incubated with goat anti-rabbit horseradish peroxidase (Dako, Golstrup, Denmark) for 40 or 60 min (ROCK1 and ROCK2/nitrotyrosine respectively) at room temperature and developed using diaminobenzidine (Vector Laboratories, Inc, Burlingame, CA, USA) and finally counter-stained with haematoxylin.

### Assessment of vessel ID

Vessel ID in individual rats was assessed as previously described [6]. Briefly, quantitative analysis of vessel ID was based on measurements from the middle of discrete vessel segments in individual cine-radiogram frames using Image-J (v1.41, NIH, Bethesda, USA) for individual rats during each treatment period. Angiograms shown in this paper underwent median filtering (2 pixel radius) to improve vessel clarity for publication purposes only. Arterial vessels were categorised according to their branching order and their basal vessel ID size class (40–100 μm, 100–200 μm, 200–300 μm and >300 μm). Reported results for vessel ID and vessel number in each rat during drug infusions are expressed as percentage change from baseline (Δ), to account for differences in absolute baseline vessel ID and vessel number between groups. Vessel recruitment was determined as the change in vessel number from baseline during each treatment for the same field of view.

### Quantification of segmental vasoconstrictions

Relative change in vessel calibre following vasodilator inhibition gives no indication of the number of vessels with calibres less than the individual’s mean change. Therefore the number of segmental vasoconstrictions during treatment periods was quantified during the treatment periods as outlined by Jenkins et al. [6]. Localised segmental vasoconstrictions were considered to be present when most of the length of a vessel segment (<100 μm) showed an ID constriction of >30% of baseline vessel ID.

### Statistical analysis

Data is expressed as mean ± SEM unless otherwise stated. The mean vessel ID and the change in ID (%) of each branching order or vessel size class in individual rats, in each treatment period, were pooled for group comparisons. One-way and two-way ANOVA with Bonferroni correction for repeated measures was performed to assess within and between group differences due to treatments. Following ANOVA, between group comparisons were made using a 2-tailed Student t-test. The Statistical Package Software System (SPSS v15, SPSS Inc, Chicago, USA) was used for all analysis with values of *P* <0.05 deemed significant.

## Results

### Animal characteristics

Diabetic animals had higher blood glucose concentrations (*P* < 0.001), lower final body weight (*P* < 0.001) and reduced heart rate under anaesthesia (*P* < 0.001) versus controls (Table 1). Kidney to BW ratio was significantly increased in diabetic animals (*P* < 0.05) while LV to BW ratio was comparable to control animals, indicating that there was no compensatory cardiac hypertrophy. Triglyceride concentration was similar in both groups.

**Table 1:** **Body and organ weights**, **metabolic profile and haemodynamic variables in anaesthetised control and diabetic Sprague**–**Dawley rats**

	**Control**	**Diabetes**	***P***
**Body Weight (BW, g)**	312 ± 10	236 ± 8	<0.001
**Blood Glucose (mmol)**	5.9 ± 0.4	19.5 ± 1.9	<0.001
**MAP (mmHg)**	116 ± 6	105 ± 7	NS
**Heart Rate (bpm)**	436 ± 7	344 ± 11	<0.001
**Triglyceride (mmol)**	0.57 ± 0.05	0.48 ± 0.21	NS
**Heart:BW (%)**	0.267 ± 0.005	0.272 ± 0.005	NS
**Kidney:BW (%)**	0.532 ± 0.019	0.820 ± 0.023	<0.001

Values expressed as mean ± SEM. Control, n = 6 and diabetic, n = 8. MAP: Mean arterial pressure.

### Structural changes and oxidative stress

**Figure 1:**
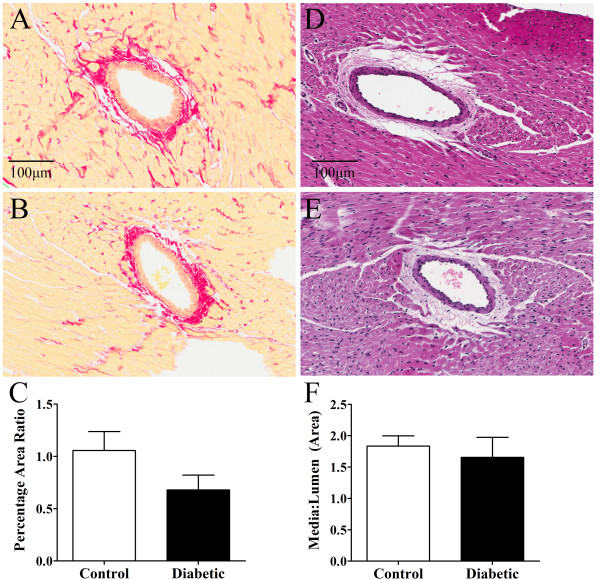
**Myocardial perivascular fibrosis and media:lumen ratio in control and diabetic rats.** Perivascular collagen ratio **(C)** was assessed via picrosirius red staining in control **(A)** and diabetic **(B)** rats (20× objective). Vessel media-to-lumen ratio **(F)** was assessed via haematoxylin and eosin staining in control **(D)** and diabetic **(E)** rats (20× objective). There was no significant difference between groups. Control, n = 6 and diabetic, n = 8. Values expressed as mean ± SEM.

**Figure 2:**
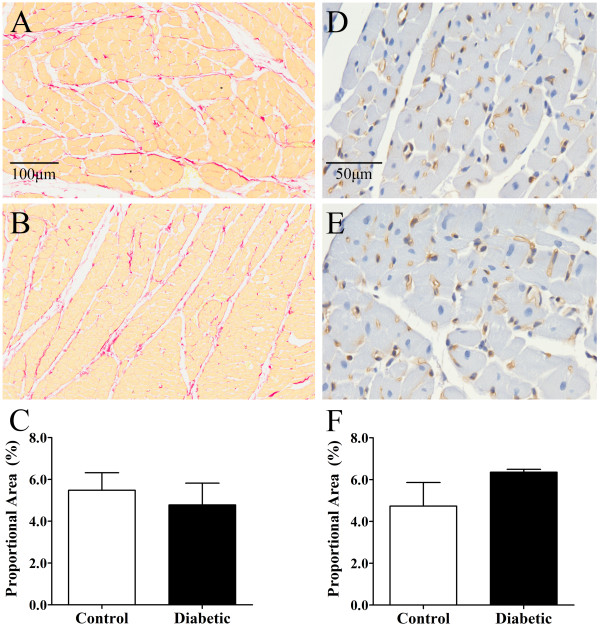
**Myocardial interstitial collagen expression and capillary density in control and diabetic rats.** Interstitial collagen accumulation **(C)** assessed via picrosirius red staining in control **(A)** and diabetic **(B)** rats (20× objective). Capillary density **(F)** assessed via isolectin B4 staining in control **(D)** and diabetic **(E)** rats (40× objective). There was no significant difference between groups. Control, n = 6 and diabetic, n = 8. Values expressed as mean ± SEM.

**Figure 3:**
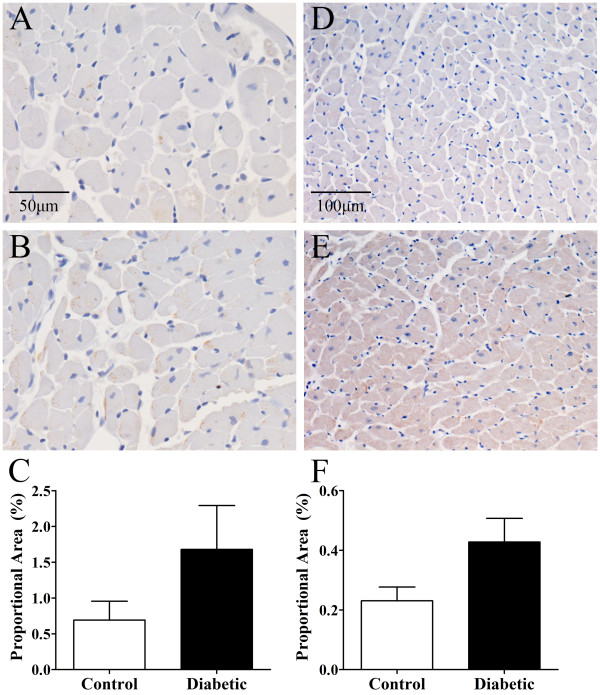
**Myocardial ROCK1 and ROCK2 immunohistochemistry in control and diabetic rats.** ROCK1 expression **(C)** in control **(A)** and diabetic **(B)** rats (40× objective). ROCK2 expression **(F)** in control **(D)** and diabetic **(E)** rats (20× objective). ROCK1 expression was not significantly different between control and diabetic groups, although there was a trend towards increased ROCK1 expression in diabetic rodents. ROCK2 expression was borderline significantly increased (*P* < 0.053) in diabetic compared to control animals. Control, n = 6 and diabetic, n = 7. Values expressed as mean ± SEM.

**Figure 4:**
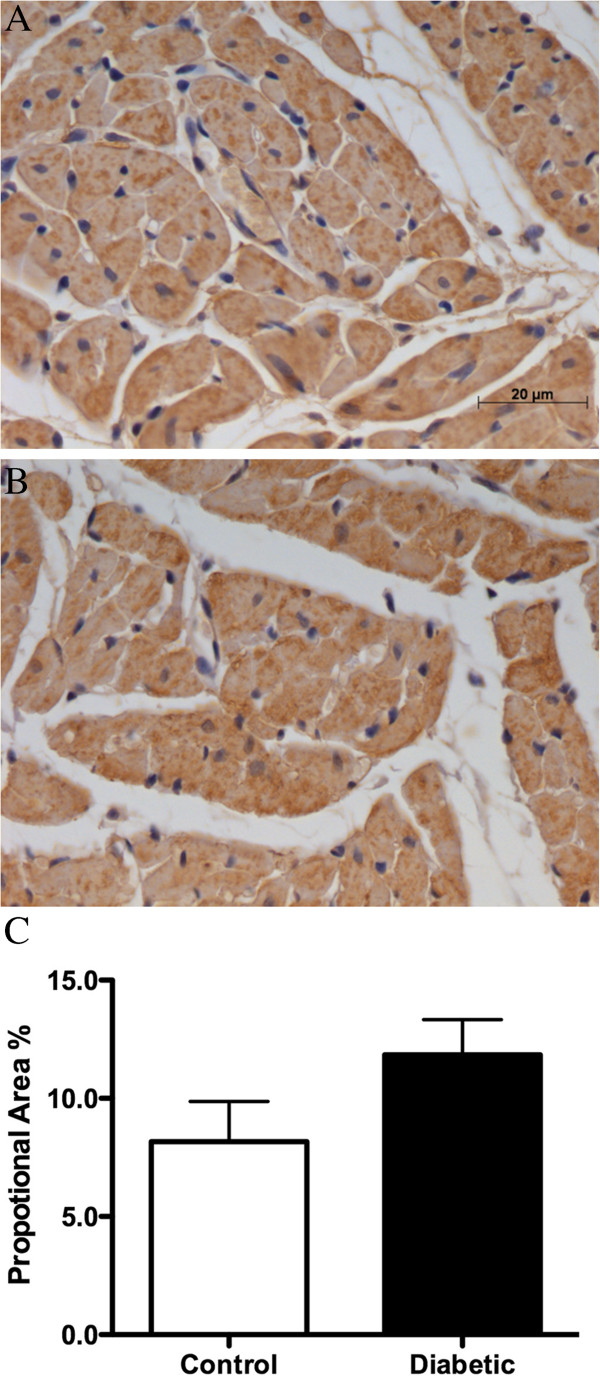
**Myocardial nitrotyrosine immunohistochemistry in control and diabetic rats.** Nitrotyrosine expression **(C)** in control **(A)** and diabetic **(B)** rats (20× objective). Nitrotyrosine expression was not significantly different between control and diabetic groups. Control, n = 5 and diabetic, n = 7. Values expressed as mean ± SEM.

Myocardial perivascular fibrosis was similar in diabetic and control rats (Figure 1A-C), as was media-to-lumen ratio (Figure 1D-F). There was also no significant difference in myocardial interstitial fibrosis between control and diabetic animals (Figure 2A-C) or capillary density (Figure 2D-F). Myocardial ROCK1 expression trended higher in diabetic versus control animals (Figure 3A-C), while myocardial ROCK2 expression was borderline significantly higher in diabetic animals 3-weeks post STZ treatment compared to controls (*P* < 0.053, Figure 3D-F). Nitrotyrosine levels did not differ significantly between groups, suggesting that diabetic rats did not experience consistently higher levels of oxidative stress (Figure 4).

### Baseline vessel internal diameter

**Figure 5:**
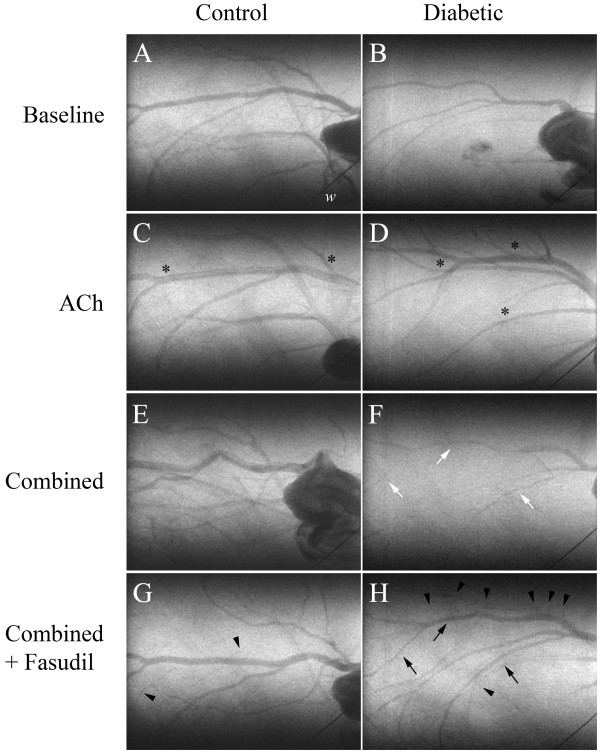
**Representative synchrotron radiation angiogram of the coronary vasculature in control and diabetic rats showing segmental constrictions.** Baseline lactate infusion **(A** &**B)**, ACh infusion **(C** &**D**; 3.0 μg/kg/min**)**, following administration of L-NAME (10 mg/kg iv. bolus) and meclofenamate **(**combined, **E** &**F**; 2 mg/kg iv. bolus**)** and subsequent combined + fasudil treatment **(G** &**H**; 10 mg/kg iv**)**. **A** &**B**, Baseline response to lactate infusion. **C** &**D**, Control and diabetic, large vessels maintained vessel ID greater than baseline (black asterisks). **E**, Control, vessel ID maintained. **F**, Diabetic segmental vasoconstriction (white arrow). **G**, Control, vessel ID maintained. **H**, Vasodilation of medium and small arteries (black arrows) and recruitment of newly visualised small arteries and arterioles relative to baseline (arrowheads). ‘w’ is a 50 μm tungsten wire for vessel ID determination.

**Figure 6:**
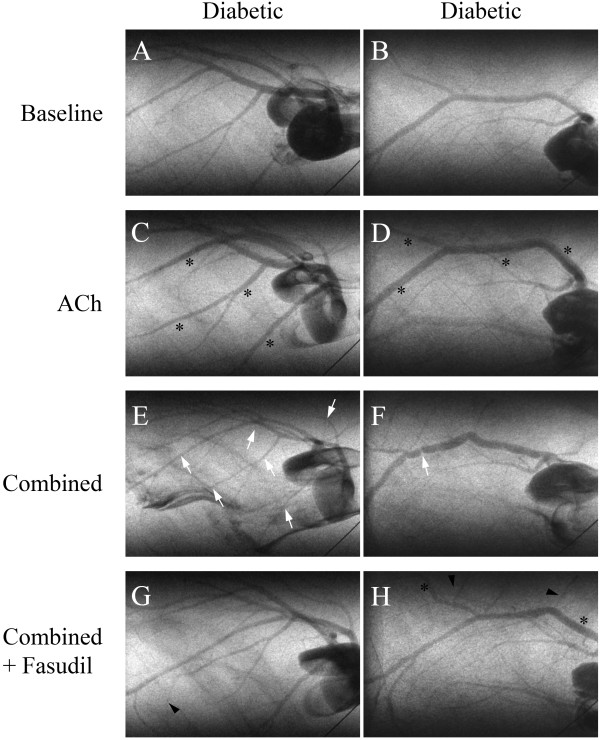
**Additional synchrotron radiation angiograms of the coronary vasculature in two diabetic rats showing segmental constrictions.** Baseline lactate infusion **(A** &**B)**, ACh infusion **(C** &**D**; 3.0 μg/kg/min**)**, following administration of L-NAME (10 mg/kg iv. bolus) and meclofenamate **(**combined, **E** &**F**; 2 mg/kg iv. bolus**)** and subsequent combined + fasudil treatment **(G** &**H**; 10 mg/kg iv**)**. **A** &**B**, Baseline response to vehicle infusion. **C** &**D**, Both diabetics maintained larger vessel ID than baseline (black asterisks). **E** &**F**, Diabetic focal and segmental vasoconstrictions (white arrows). **G** &**H**, Restoration of medium and small arteries to baseline ID, dilation (black asterisks) and recruitment of newly visualised small arteries and arterioles relative to baseline (arrowheads). ‘w’ is a 50 μm tungsten wire for vessel ID determination.

**Figure 7:**
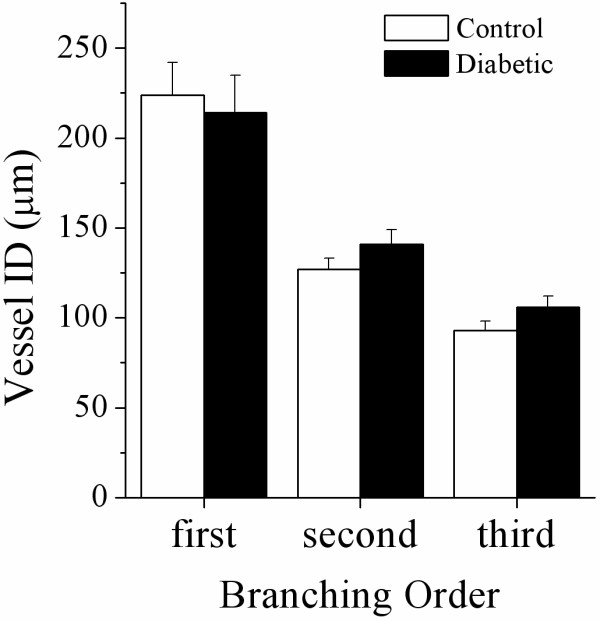
**Vessel internal diameter categorised by branching order in control and diabetic animals during baseline vehicle infusion.** There was no significant difference between control and diabetic animals. Control, n = 6 and diabetic, n = 8. Values expressed as mean ± SEM.

Representative angiogram frames for all treatment periods are presented in Figures 5 and 6 for a control and STZ rats to illustrate the limited individual variability in responses. Basal vessel ID in diabetic animals was comparable to control vessel ID across first (214 ±21 vs. 224 ±18 μm), second (141 ±8 vs. 127 ±6 μm) and third (106 ±6 vs. 93 ±5 μm) order arterial vessels (Figure 7). Mean total visible vessel branching segments per animal in the field of view, was 12–18 vessels across all treatments in control animals and a comparable 11–14 vessels in diabetic rats. The minimum visualised vessel ID recorded across both groups was 44 μm.

### Vessel response to endothelium-dependent and -independent stimulation

**Figure 8:**
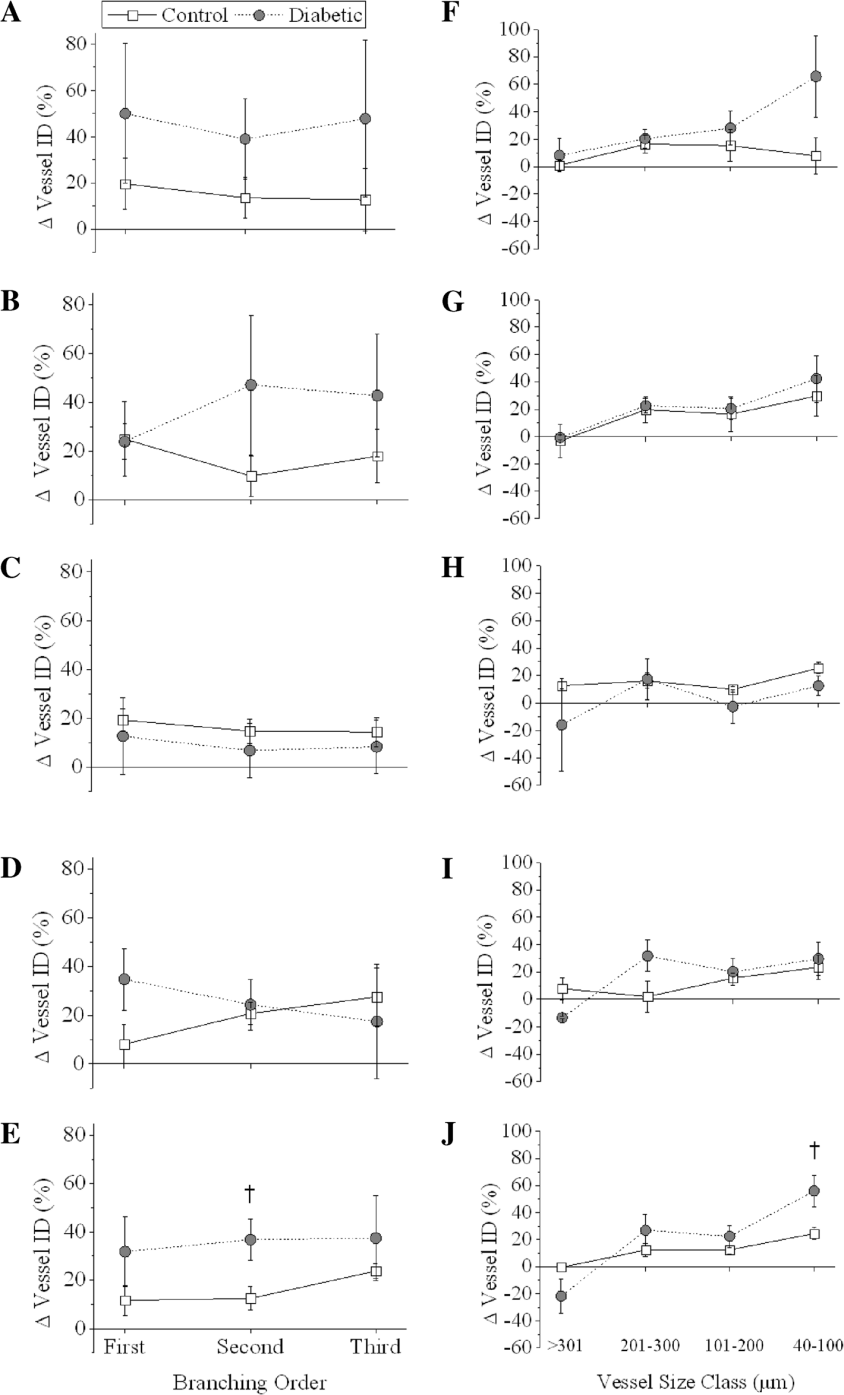
**Change in vessel ID in control and diabetic animals during infusion of vasoactive compounds**, **categorised by branching order and vessel size class.** ACh **(A** &**F**; 3.0 μg/kg/min**)**, SNP **(B** &**G**; 3.0 μg/kg/min**)**, combined **(C** &**H)**; L-NAME (10 mg/kg iv. bolus) + meclofenamate (2 mg/kg iv. bolus), combined + ACh **(D** &**I)** and combined + fasudil **(E** &**J**; 10 mg/kg iv.**)**. Fasudil treatment resulted in a marked increase in diabetic second order vessel ID and the 40-100 μm small artery-arterioles compared to controls. Control, n = 6 and diabetic, n = 8. Values expressed as mean ± SEM. †*P* < 0.05 vs. control.

**Figure 9:**
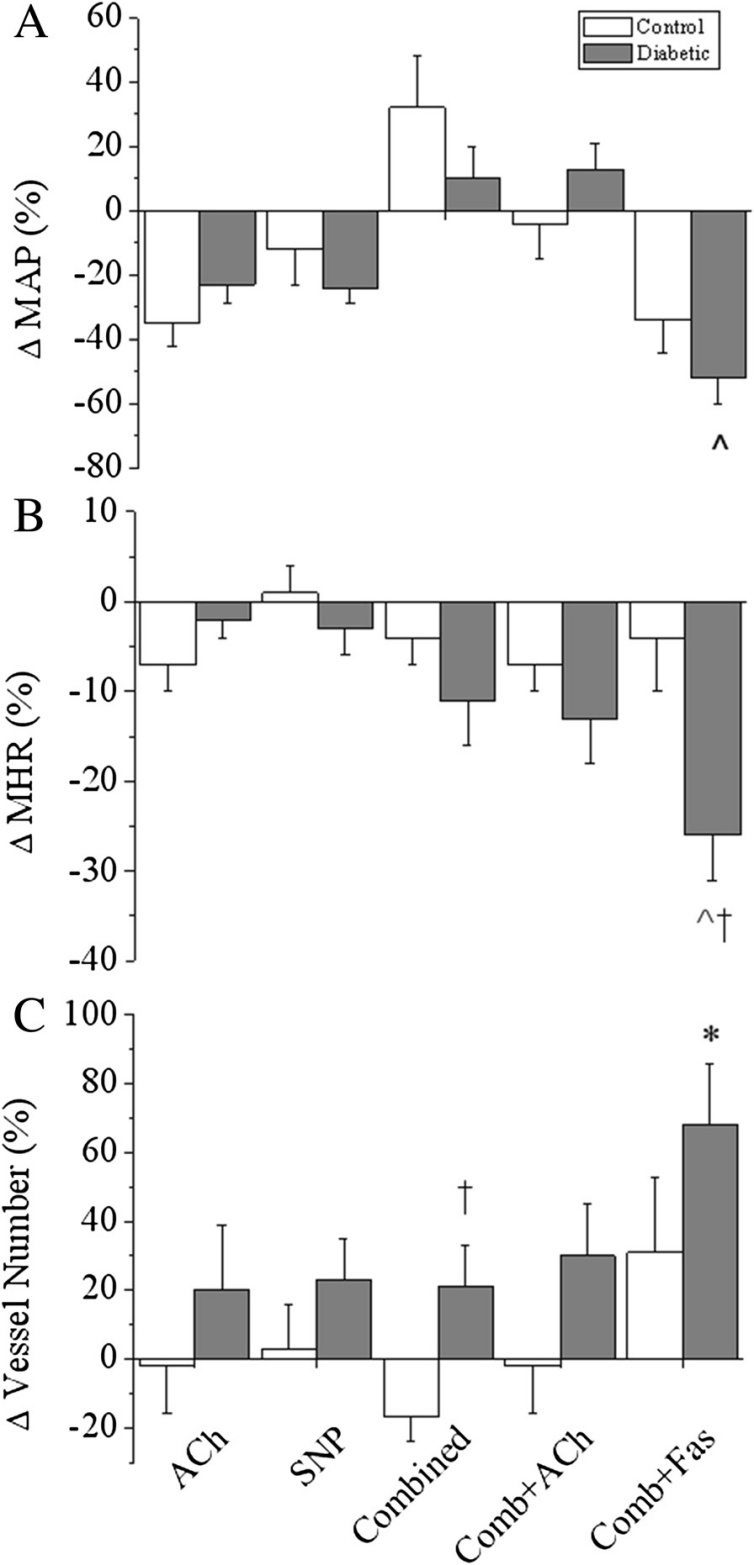
**Mean arterial pressure (A), heart rate (B) and overall vessel recruitment (C) in control and diabetic rats.** Changes were assessed during infusion of ACh (3.0 μg/kg/min), SNP (3.0 μg/kg/min), combined; L-NAME (10 mg/kg iv. bolus) + meclofenamate (2 mg/kg iv. bolus), combined + ACh and combined + fasudil (10 mg/kg iv.) relative to baseline (lactate 3.0 ml/hr). **A**, Diabetic rats had a significant decrease in blood pressure during combined + fasudil treatment compared to baseline. **B**, Heart rate was significantly reduced in diabetic animals during fasudil treatment compared to baseline and control rats. **C**, Diabetic rats had significantly greater vessel recruitment during combined NOS/COX blockade compared to control animals. Control, n = 6 and diabetic, n = 8. Values expressed as mean ± SEM. *P < 0.05, ^*P* < 0.001 vs. baseline. †*P* < 0.05 vs. control.

Basal endothelium-dependent vasodilation in diabetics in response to ACh was on average more pronounced (>40% of basal ID) than in control rats, but not significantly so, based on vessel order or vessel class (Figure 8A and F). In response to ACh, similar reductions in MAP (Δ -23.3 ±6.0% vs. -31.4 ±6.5%) and HR (Δ -6.87 ±3.09% vs. -2.21 ±2.38%, Figure 9) were observed in both groups. Basal endothelium–independent dilatory responses to SNP were comparable between diabetic and control animals (Figure 8B and G). In addition, the MAP and HR response to SNP was not significantly different between groups (Figure 9).

### Vessel ID response during vasodilator inhibition

Combined blockade of NOS and cyclooxygenase (COX) caused vasodilation of the coronary vasculature in both control and diabetic animals. This was evidenced by a slightly greater vessel ID in both groups compared to baseline (Figure 8C and H). However, diabetic animals had a slightly blunted, but not significant, increase in MAP in response to NOS/COX blockade (Δ 9.7 ±9.7% vs. 31.9 ±16.4%, Figure 9). HR was reduced by similar amounts in control and diabetic animals following NOS/COX blockade. Vessel ID response to ACh stimulation during NOS/COX blockade was not different between control and diabetic rats (Figure 8D and I). Further, MAP in control and diabetic animals returned to baseline levels, while there was a small but persistent reduction in HR in both groups (Figure 9).

### Vessel ID response to fasudil treatment

Inhibition of ROCK during NOS/COX blockade resulted in a significantly greater vessel ID response in second order vessels in diabetic animals compared to controls (Δ 36.7 ±8.5% vs. 12.4 ±5.0%, *P* < 0.05, Figure 8E). This difference in vessel ID, based on branching order, is also borne out when vessels were categorised by vessel class, as fasudil treatment caused a marked increase in vessel ID in 40-100 μm vessels from diabetic animals compared to controls (*P* < 0.05, Figure 8J). There was a trend in both control and diabetic groups towards a progressively greater vasodilatory response to fasudil as vessel size class decreased. The greater increase in microvessel ID in diabetic animals did not correspond with a significantly greater reduction in MAP from baseline in comparison to control rats (Δ -51.8 ±2.6% vs. -34.5 ±9.5%, *P* < 0.001, Figure 9). HR was significantly reduced compared both to baseline and control animals in response to fasudil in diabetic animals (*P* < 0.05, Figure 9).

### Vessel recruitment during fasudil treatment

The number of vessels visible during ACh or SNP-mediated vasodilation was not significantly different between control and diabetic rats. During combined NOS/COX blockade vessel recruitment was significantly greater in diabetic animals compared to controls (Δ 19.9 ±10.8% vs. -12.7 ±7.8%, *P* < 0.05, Figure 9). Following stimulation with ACh, in the presence of combined blockade, this difference in recruitment was reduced. Vessel recruitment in response to fasudil was markedly increased in diabetic animals compared to baseline (*P* < 0.05), however this was not significantly different to control animals.

### Effect of fasudil on vascular constrictions

**Figure 10:**
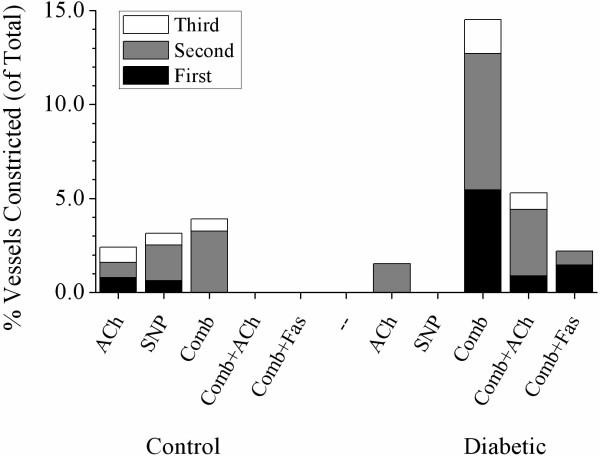
**The occurrence of segmental constrictions classified by branching order in control and diabetic rats.** Assessed during administration of ACh (3.0 μg/kg/min), SNP (3.0 μg/kg/min), combined; L-NAME (10 mg/kg iv. bolus) + meclofenamate (2 mg/kg iv. bolus), combined + ACh and combined + fasudil (10 mg/kg iv.). Segmental constrictions predominately occurred in second order vessels in control animals. In diabetic animals constrictions were predominant in second order vessels and to a lesser extent in first order. ACh administration following combined NOS/COX blockade alleviated first order constrictions while fasudil treatment further reduced second order constrictions. Segmental constrictions are expressed as a proportion of total vessels visible. Control, n = 6 and diabetic, n = 8. Values expressed as mean ± SEM.

One focal constriction (>70% of basal ID) was observed in a control and a diabetic rat (Figure 5F, white arrow) during combined blockade, but was abolished by ACh infusion in the control rat only. Subsequent fasudil treatment was able to completely eliminate the remaining focal constriction in the diabetic rat. Segmental constrictions were more prevalent in diabetic animals, following combined blockade (Figure 5F and 6E; white arrows and Figure 10), although this was not significant compared to control animals. Constrictions occurred predominantly in the first and second order vessels from diabetic animals (Figure 10). Consistent with the focal constriction findings, ACh administration during combined blockade completely eliminated all segmentally constricted vessels in control but not diabetic animals (Figure 10). Following fasudil treatment, segmental constrictions were markedly reduced in diabetic animals (Figures 5H and 6G and H; black arrows) compared to NOS/COX blockade alone, notably in second and third order vessels.

## Discussion

This study has demonstrated a widespread involvement of the ROCK signalling pathway in early stage diabetic coronary dysfunction *in vivo*. This is supported by data showing that acute ROCK inhibition alleviates focal and importantly segmental coronary constrictions in early diabetes. Furthermore, acute ROCK inhibition in the diabetic heart results in a significant increase in both micro- and macrovessel calibre in second and third order branching segments, following NOS/COX inhibition. This role for ROCK activation occurs prior to impairment in endothelium-dependent vasodilation, coronary structure or the ability of the coronary circulation to recruit microvessels during blood flow increases.

The three-week post diabetes induction time point used in this experiment, thus allows the investigation of early functional changes in the diabetic vasculature prior to changes in coronary structure, which are known to occur in the later stages of diabetes [24-26]. The extent of perivascular fibrosis, media-to-lumen ratio and capillary density were similar in diabetic and control animals and furthermore were consistent with our previous findings [6]. In addition, the likelihood that myogenic, metabolic or endothelial responses differentially affect coronary tone is limited, as coronary driving pressures were similar in diabetic and control animals during all treatments.

### Vascular function in the presence of nitric oxide and prostacyclin

Basal coronary endothelial function in diabetic rats was maintained at 3 weeks post induction, however there are reports from experiments in isolated coronary arteries suggesting that by 4 weeks, endothelium-dependent vasodilation is impaired [27,28]. Consistent with the current findings, in our previous study, although there were early indications basal coronary function was starting to deteriorate after 3 weeks of diabetes [6], coronary vasodilation *in vivo* was actually maintained. This preserved coronary endothelium-dependent vasodilation, may be due to a compensatory increase in coronary COX-2 activity and expression as demonstrated in early stage diabetic mice [29]. It may also result from an increased expression of eNOS, which was found in carotid arteries in early diabetes, although this is less likely as the increase in activity is also associated with greater eNOS uncoupling [30]. Notably, vascular smooth muscle function in diabetic animals was well maintained as evidenced by similar endothelium-independent vasodilation when compared to control animals.

### Basal EDHF function and reserve

Vasodilatory responses were similar post-NOS/COX blockade across all branching orders and vessel classes in diabetic and control animals although the ability of the diabetic coronary circulation to recruit new vessels was slightly enhanced. This increase in visible perfused segments likely results from an acute compensatory response to maintain coronary blood flow following NOS/COX blockade. Endothelium stimulation following combined blockade facilitates the assessment of the ability of the remaining endogenous vasodilators namely, EDHF, to maintain vasodilation. These findings agree with our previous study [6], in suggesting that global coronary EDHF reserve is unchanged in this model of diabetes, as EDHF pathways remain intact and capable of providing sufficient reserve to overcome any vasoconstrictor influences.

### Vessel diameter response to fasudil

To assess the role of ROCK in the diabetic coronary circulation *in vivo*, this study utilised the potent and selective ROCK inhibitor fasudil, due to its minimal off-target effects on related signalling pathways, including myosin light chain kinase and protein kinase C (PKC) [31]. Fasudil is non-selective for the isoforms of ROCK, ROCK1, expressed ubiquitously, except in brain and muscle tissue and ROCK2, expressed primarily in cardiac and brain tissue [32]. However, it is unlikely that the limited time of fasudil treatment in the current study would be sufficient to result in its significant conversion to the more highly potent and selective, hydroxyfasudil [18] and as such some of the inhibitory effect of fasudil may result in part from off-target inhibition of other kinases. Importantly, in the context of cardiovascular disease and diabetic coronary dysfunction, both ROCK1 and ROCK2 are expressed in vascular endothelial and smooth muscle cells [33-35].

In the diabetic coronary microcirculation ROCK inhibition resulted in significantly greater vasodilation compared to controls that was most evident in the small arteries and arterioles emerging from the epicardial main artery segments. Hence, acute fasudil treatment increases global perfusion in the early diabetic heart. Consistent with these findings, Didion et al. have shown that ROCK inhibition significantly increased vessel diameter of cerebral arteries from type-2 diabetic db/db mice but not in controls [20]. Moreover, fasudil has been shown to reverse vasoconstriction in more advanced diabetes [33,36].

### Focal and segmental constrictions in diabetes

In the early diabetic state, prior to widespread vessel impairment in coronary endothelium mediated vasodilatation, we have characterised and quantified focal and segmental constrictions in the microvasculature [6]. This study also revealed a strong trend towards an increased incidence of segmental constrictions in diabetic animals when there is a deficit in vasodilator production. This suggests that in later stage diabetes, when the actions of NO and PGI_2_ are progressively impaired [37], the coronary circulation may become more vulnerable to constrictions. Therefore, further assessment of the role these constrictions play in the regulation of coronary perfusion in diabetes remains vital.

Endothelium-dependent stimulation post-NOS/COX blockade abolished all segmental constrictions in control but not diabetic animals. More detailed analysis showed that the segmental constrictions occurred predominantly in epicardial first order vessels in diabetic animals. This is somewhat unexpected as small alterations in vascular tone, at this macrovessel level are less common, as they can have significant effects on downstream microvascular perfusion and are actually predictive of longer term cardiovascular disease risk in type-2 diabetes [38].

### A role for ROCK in focal and segmental constrictions

A novel finding in this study, is the *in vivo* identification of ROCK as a likely mediator of localised constrictions in diabetic hearts. This is supported by the fact that acute ROCK inhibition greatly reduced the incidence of segmental constrictions following NOS/COX blockade. Consistent with this, ROCK has previously been shown to be upregulated in non-diabetic porcine hearts at the site of coronary artery spasm [39]. Hypercontraction by ROCK activation and increased myosin binding subunit phosphorylation in vascular smooth muscle cells appears to be a key to much vascular dysfunction. Localised ROCK activation has been implicated in cerebral artery vasospasm following subarachnoid haemorrhage [40], coronary artery spasm [10], and following coronary artery bypass [41] and myocardial ischemia [17]. In our study, non-constricted vascular regions in diabetic animals responded to ROCK inhibition similarly to that of responses in control animals, in agreement with findings in the human coronary circulation where ROCK inhibition had minimal effect on non-spastic segments [42]. This suggests that during early-stage diabetes there remain vessel segments where changes in ROCK signalling are not contributing appreciably to vasomotor responses.

Immunostaining for ROCK1 expression in the myocardium and endothelium was not significantly different between diabetic and control animals although there was a notable trend towards an increase. While not significant, our results are in line with previous work which showed significant ROCK1 expression upregulation in thoracic aortas from 3-week diabetic rats [43]. ROCK2 expression was borderline significantly increased in our diabetic animals, as reported in retinal vessels from rats 2 weeks after inducing diabetes [15]. Irrespective of ROCK expression levels in the early diabetic heart a functional role for ROCK activation was clear.

Diabetes is known to increase RhoA expression, the upstream regulator of ROCK, as shown in the basilar artery membrane at 2 weeks [44] and aortic homogenates at 4-weeks post STZ [28] in diabetic rodents. Furthermore, RhoA expression remains elevated in more advanced diabetic stages, as described in aorta from 12–14 week old [13] and 12–40 week old [33] db/db mice. However, to date the effect of diabetes on coronary ROCK expression and activity in more advanced stages remain unclear. Longer term type-2 diabetes studies using aortic and mesenteric arteries suggest that ROCK expression is unchanged [33,36], although whether this is true in the coronary circulation is uncertain. It remains to be determined if RhoA upregulation drives ROCK activation and localised coronary constrictions in early diabetes.

### Possible mechanisms for ROCK-mediated vasoconstriction

There is strong evidence that hyperglycaemia and or hyperlipidaemia evoke reactive oxygen species to stimulate RhoA/Rho-kinase signalling [45]. Remnant lipoproteins, acting via the RhoA/Rho-kinase pathway to cause coronary vasospasm are considered to be a major factor in sudden death in humans [46]. Nevertheless, our findings suggests that lipids may have a minimal role in the initiation of ROCK-mediated vasoconstriction in our diabetic animals, as plasma triglyceride concentrations were comparable to control animals, contrasting with later diabetic states [27,47]. Furthermore, the absence of a significant widespread increase in nitrotyrosine levels in the coronary vessels and myocardium of diabetic rats in this study suggests that oxidative stress is not yet contributing appreciably to ROCK-mediated vasoconstriction at this time point. Plasma triglycerides and nitrotyrosine are only single measures of lipid accumulation and oxidative stress, therefore further assessment of lipid profiles and other reactive oxygen and nitrogen species are certainly warranted.

Hyperglycaemia and diacylglycerol accumulation are well known for inducing activation of multiple PKC isoforms, and in particular PKCβ, to cause vascular complications and potentiated vasoconstrictor responses in diabetic coronary arteries [48]. While PKC mediated potentiation of ET_1_ constrictor tone is implicated, diabetic myocardial contractile impairment has been shown to be a consequence of PKC activation of downstream inducible NOS/RhoA/ROCK [49-51]. Kizub et al. also suggest that both ROCK and PKC activity evoke enhanced myofilament Ca^2+^ sensitivity in muscular arteries in diabetes [52]. It is important to note however that ROCK inhibition did not completely alleviate all diabetic segmental constrictions, suggesting that although important, ROCK is unlikely to be the sole mediator of the elevated diabetic coronary constrictor tone.

### Experimental limitations

In comparison to our previous study of early diabetes [6], the extent of microvascular dysfunction, both focal and segmental constriction, was slightly less advanced in this cohort of diabetic animals. This was also the case with the attenuation of endothelium-dependent vessel recruitment, which in this study, was not significantly different from controls. This may be due to a slower development in the pathogenesis of diabetes. Indeed, although the average blood glucose concentration was greater in the current study, this was also coupled with greater between animal variability in BG (12.7- 27.9 mmol/L this study vs. 16.7-19.4 mmol/L) [6]. Some diabetic animals in the current study may have initially had lower blood glucose concentrations for part of the 3-week period after STZ induction. This could have led to a shortened period of severe hyperglycaemia and the partial maintenance of coronary microvascular function.

A second limitation of this study that needs to be recognised is that we cannot accurately determine global coronary flow or velocity in the anaesthetised rat heart at this time with the SATICON detector video frame rates [53]. The advantages of SR microangiography, including increased spatial and temporal resolution, far outweigh this limitation, and allows the assessment of coronary microvessel diameter, and therefore local distribution of vascular resistance and function *in vivo*[6,53].

### Future directions

Future investigations may focus on treating diabetic cardiomyopathy with ROCK inhibitors. Indeed recent evidence in diabetic rodents suggests that acute ROCK inhibition also corrects myocardial contractile function [49,54]. Fasudil treatment has also been shown to preserve the post-conditioning capacity of the heart during hyperglycaemia and protect against myocardial infarction through the opening of mitochondrial ATP-sensitive K channels [55]. Furthermore, others have shown that inhibition of ROCK reduces monocyte cell adhesion to the endothelium [56], an early event in plaque formation, and reduces early plaque formation and the size of established plaques [57]. Since ROCK is an early contributor to coronary vascular dysfunction, by increasing constrictor tone and subsequently endothelial dysfunction, then chronic treatment with ROCK inhibitors may help prevent the diabetic coronary dysfunction completely. A potential added benefit of this therapy is likely to be a reduction in diabetic vascular complications associated with both vascular inflammation and the pro-atherogenic state in diabetics [56].

## Conclusions

In summary, we have confirmed that *in vivo* imaging using SR is a powerful means of investigating the intact coronary circulation. Furthermore, we have shown *in vivo*, that in early-diabetic rats increased ROCK activity plays a role in coronary microvasculature function, as acute ROCK inhibition increased coronary perfusion. Notably, ROCK-mediated vasoconstriction was not a consequence of increased oxidative stress, inferring that it was most likely mediated by increased activation of myosin light chain phosphatase. However, at this early diabetic stage the ROCK mediated increase in vasoconstrictor tone is normally offset by NO and PGI_2_. Nevertheless, these findings highlight the potential importance of the ROCK signalling pathway in diabetic coronary vasculature and provide a promising target for future therapeutic interventions.

## Abbreviations

ACh: Acetylcholine; BG: Blood glucose; BW: Body weight; COX: Cyclo-oxygenase; EDHF: Endothelium derived hyperpolarising factors; eNOS: Endothelial nitric oxide synthase; ET1: Endothelin 1; HR: Heart rate; ID: Internal diameter; L-NAME: Nω-nitro-l-arginine methyl ester; LV: Left ventricle; MAP: Mean arterial pressure; NO: Nitric oxide; NOS: Nitric oxide synthase; PGI2: Prostacyclin; PKC: Protein kinase C; ROCK: RhoA/Rho-kinase; SEM: Standard error of the mean; SNP: Sodium nitroprusside; SR: Synchrotron radiation; STZ: Streptozotocin.

## Competing interests

The authors declare that they have no competing interests.

## Authors’ contributions

JTP, MJJ and MS participated in the design of the study, carried out the imaging experiments and contributed to the data analysis and writing of the manuscript. AJE participated in the imaging studies, oversaw and optimised immunohistochemistry. TS, MJJ, DOS, YF and HT prepared animals and collected physiological data for the imaging experiments. MJJ, MW and DJK contributed to histology and immunohistochemistry preparation and analysis and supervision of these analyses. All authors read and approved the final manuscript.
